# Fine needle aspiration cytology diagnosis of paravertebral extraosseus Ewing’s sarcoma

**DOI:** 10.4103/0970-9371.73304

**Published:** 2010-10

**Authors:** Archana C Buch, NK Panicker, Sweta Sarawagi, Sampada Anwekar, Amit T Kharat

**Affiliations:** Department of Pathology, Padmashree Dr. D.Y. Patil Medical College, Pimpri, Pune - 411 020, India; 1 Department of Radiology, Padmashree Dr. D.Y. Patil Medical College, Pimpri, Pune - 411 020, India

**Keywords:** Extraskeletal Ewing’s sarcoma, fine needle aspiration cytology, paravertebral region

## Abstract

Extraskeletal Ewing’s sarcoma (EES) is a rare tumor. Paravertebral Ewing’s sarcoma requires more extensive therapy as compared to Ewing’s sarcoma of bone. Fine needle aspiration cytology (FNAC) plays an important role in the early diagnosis of these cases. We present a case where paravertebral extraosseous Ewing’s sarcoma was diagnosed on FNAC in a 19-year-old girl.

## Introduction

Ewing’s sarcoma of bone (ES), extraskeletal Ewing’s sarcoma (EES) and primitive peripheral neuroectodermal tumor (pPNET) are now considered to be the same tumor with variable differentiation and no significant biological or therapeutic distinction.[1] With a change in treatment modality of Ewing’s-pPNET family of tumors by preoperative chemotherapy, fine needle aspiration cytology (FNAC) plays a useful role in the diagnosis of these lesions with obvious advantage over the open/needle core biopsy.

EES has been described in different locations such as larynx, scalp, nasal fossa, neck, chest wall, lung, perineum, finger, arm, lip and toe. EES requires more extensive therapy as compared to ES. It is essential to diagnose these cases early to change clinician’s traditional method of handling such a case. We report one such case of paravertebral EES diagnosed by ultrasound guided FNAC.

## Case Report

A 19-year-old girl was admitted with complaints of progressively increasing dull aching pain in the lower back and right lower limb since 2 months. There was a history of loss of appetite, mild fever and malaise. The patient was thin built. On general examination, there was a mild pallor. Systemic examination did not reveal any abnormalities, except weakness (power 3/5) in the right lower limb.

Investigations revealed hemoglobin of 10 g%, total leucocyte count of 9200 cells/mm^3^ with 75% polymorphs and 23% lymphocytes. Erythrocyte sedimentation rate (ESR) was 65 mm at the end of 1 hour. The urine examination for albumin, sugar, vinyl mandelic acid and the bone marrow examination did not reveal any abnormalities. Tuberculin test was negative. Chest radiograph was normal. Radiograph of the spine revealed a radiopaque soft tissue mass in right paravertebral region, measuring 8×7 cm, in L5-S1 region. Ultrasound revealed a large ill-defined echo-poor heterogeneous lesion in the right paraspinal region.

**Figure 1:**
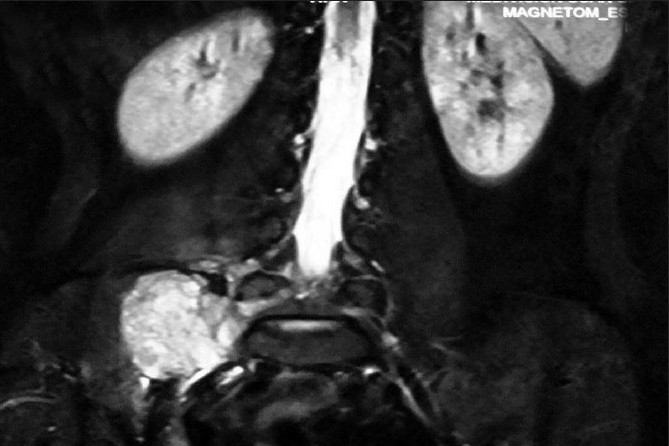
T1-weighted MRI showing a tumor mass at L5-S1 region

Magnetic resonance imaging (MRI) scan revealed relatively well-defined, lobulated, heterogeneously enhancing soft tissue lesion with large paraspinal component encroaching spinal canal through the right L5-S1 foramina, causing a mass effect [Figure 1]. Associated altered signals and the pressure erosions over the adjacent bones due to infiltration and inflammatory reaction were noted. The lesion was suspected to be of neurogenic origin. FNAC was advised for definite diagnosis. USG guided FNAC was done using long 22 gauge disposable needle and 10 ml syringe. Slides were prepared and stained with May-Grünwald-Geimsa (MGG). Two slides were reserved for special stains and studies.

**Figure 2:**
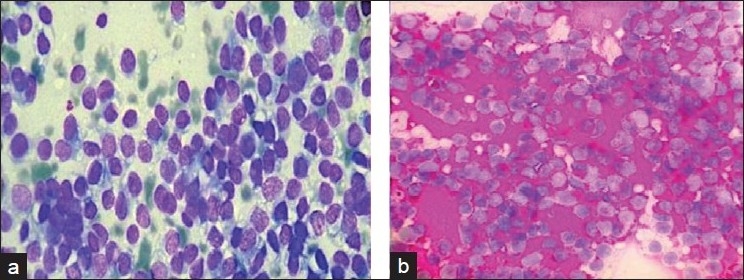
(a) EES shows clusters of monomorphic tumor cells with vacuolated cytoplasm (MGG, ×400), (b) Tumor cells showing cytoplasm positivity with PAS (PAS, ×400)

FNAC revealed cellular smears composed of densely dispersed, small, monomorphic round cells with fine nuclear chromatin and round nuclei and scanty clear cytoplasm [Figure 2a]. Occasional mitoses were noted at places. Many cells showed irregularly vacuolated cytoplasm which on periodic acid Schiff (PAS) stain revealed intense PAS positivity [Figure 2b]. Focal areas showed striking clusters, occasional rosette-like formations and moulding of nuclei. The background was clean.

**Figure 3:**
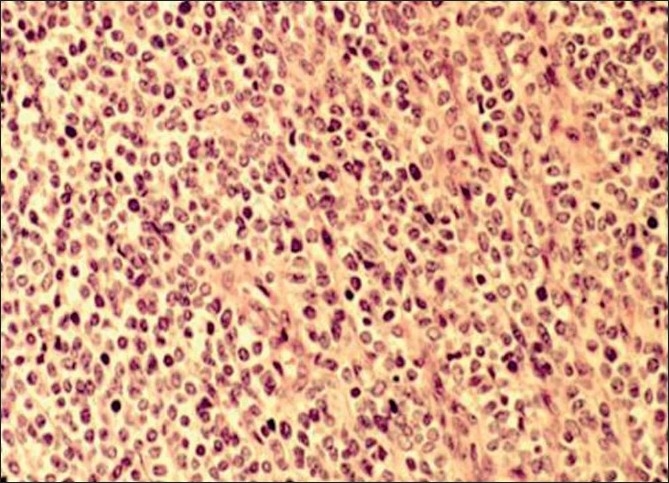
Photomicrograph showing sheets of monomorphic small round blue cells with scant cytoplasm (H and E, ×400)

FNAC diagnosis of malignant small round cell tumor, most likely ES, was offered. Later, a core biopsy was performed for confirmation. Histopathological sections revealed tumor with solidly packed round cell pattern of striking uniformity. The individual cells possessed a round or ovoid nucleus measuring 10 *μ*m in diameter with a distinct nuclear membrane, finely divided powdery chromatin, and a single minute nucleolus. The cytoplasm was scanty and pale staining. PAS stain revealed positive intracellular deposits of glycogen. Psuedorosettes were also noted. Vascularization was prominent [Figure 3]. The patient was referred to tertiary care centre for further management. Immunohistochemistry was performed there, in which the tumor cells exhibited nonspecific enolase (NSE) and vimentin along with diffuse membranous staining for CD-99. The patient was given multiagent chemotherapy combined with en bloc resection and radiotherapy.

## Discussion

EES is a poorly differentiated, highly malignant, round cell tumor without cellular or structural differentiation, and aggressive clinical behavior with high rate of local recurrence and distant metastasis.[2,3] The reported common sites of origin are trunk (paravertebral region and chest) (32%), extremity (26%), head and neck (18%), retroperitoneum, pelvis (16%), and others (10%).[2]

The origin of EES and ES has been a matter of research since the time James Ewing described the first case of ES in 1921.[3] EES was first described by Tefft *et al*,[4] in 1969. In 1975, Angerwall and Enzinger[5] reviewed 39 cases of EES resembling ES. In general, the tumor presents as a rapidly growing, deeply located mass measuring 5–10 cm in greatest diameter. The tumor is painful in about one-third of cases. If peripheral nerve or spinal cord is involved, there may be progressive sensory or motor disturbances. As with other round cell sarcomas, the preoperative duration of symptoms is usually less than 1 year. Unlike neuroblastoma, catecholamine determinations are within normal limits. Radiographs, computed tomography (CT) scan or MRI is essential for establishing the extraskeletal site of the tumor. Cytology and histopathology are essential for definite diagnosis.

Ewing’s sarcoma is richly vascular and shows an abundance of thin-walled vessels within the fibrovascular septae. The vessels tend to get compressed and obscured by closely packed tumor cell population. This cytomorphological observation may prove to be a useful clue to cytological diagnosis.[6]

ES represents the most undifferentiated, and the pPNET, the most differentiated ends of the spectrum. The two ends differ in morphology as well as in neuronal differentiation. This family of tumors shares common cytogenetic and molecular changes which involve the translocation of Ewing’s sarcoma gene on chromosome 22 (22q12) on to a number of other genes like *fli-1* on chromosome 11 (11q24) in 90% of cases and *erg* on chromosome 21 (21q22).[7] The tumors also share expression of glycoprotein surface antigen p30/32 (mic2)/CD99, a cell membrane protein of unknown function.

The differential diagnosis on FNAC and histopathology includes other round cell tumors like metastatic neuroblastoma, rhabdomyosarcoma and non Hodgkin’s Lymphoma (NHL) in children, and metastatic small cell anaplastic carcinoma and NHL need exclusion in adults.

The divisions of these tumors into skeletal and extraskeletal tumors appears more relevant than subtyping into ES, EES and pPNET. The skeletal Ewing sarcoma is known to have a 5-year survival of 75%.[8] The EES and pPNET, on the other hand, have 38% survival.[9] Paravertebral EES patients may require more extensive field radiation and more intensive chemotherapy to achieve better local and systemic tumor control.[10]

## Conclusions

FNAC is a very useful, economic and quick procedure in the diagnosis of Ewing sarcoma family of tumors. Accurate diagnosis can be made even in deep seated tumors as in our case, if one gets adequate material under radiological guidance. One needs biopsy for confirmation if scanty material is obtained. FNAC is also useful in long-term follow-up of these cases for diagnosis of recurrence or rarely intercurrent second malignancy.[11]
